# Extensive Craniofacial Trauma Caused by an Intraoral Pyrotechnic Explosion in a Patient with Paranoid Schizophrenia

**DOI:** 10.3390/jcm15197618

**Published:** 2026-10-01

**Authors:** Aleksandra Gnaczyńska, Małgorzata Król, Patrycja Kupnicka, Olaf Bieschke, Eryk Prajwos, Antoni Krężel, Joanna Andrzejak, Sascha Alexander Pietruschka, Katarzyna Barczak

**Affiliations:** 1 Department of Endodontic Surgery, Pomeranian Medical University in Szczecin, Powstańców Wlkp. 72 St., 70-111 Szczecin, Polandjoanna.andrzejak@pum.edu.pl (J.A.); katarzyna.barczak@pum.edu.pl (K.B.); 2 Department of Biochemistry, Pomeranian Medical University in Szczecin, al. Powstańców Wlkp. 72, 70-111 Szczecin, Poland; 3 Department of Radiology and Neuroradiology, Dietrich-Bonhoeffer-Klinikum, Allendestrabe 30, 17036 Neubrandenburg, Germany; olafleobieschke@gmail.com (O.B.);; 4 Department of Interdisciplinary Dentistry, Pomeranian Medical University in Szczecin, al. Powstańców Wlkp. 72, 70-111 Szczecin, Poland

**Keywords:** craniofacial trauma, firework, mandibular fracture, midface fracture, paranoid schizophrenia

## Abstract

**Background**: Intraoral explosions of pyrotechnic devices are exceptionally rare and combine blast overpressure, penetrating fragments, thermal injury, and extensive wound contamination. The immediate threats include airway obstruction, hemorrhage, loss of facial buttresses, and destruction of the occlusal unit. This report describes the acute multidisciplinary management of a self-inflicted intraoral pyrotechnic explosion in a male patient during an exacerbation of paranoid schizophrenia. **Case presentation**: The explosion caused bilateral comminution of the midface, fractures of the mandibular body/symphysis and right condylar process, a right orbital floor defect, extensive injury to the palate and tongue, bilateral wounds of the oral commissures and cheeks, and retention of metallic and pyrotechnic debris. Concomitant polytrauma included spinal, pelvic, rib, and pulmonary injuries. Massive edema and oral bleeding necessitated emergency tracheostomy. The otolaryngology team reconstructed the tongue and floor of the mouth, after which the maxillofacial team performed prolonged skeletal reconstruction through combined external and intraoral approaches. The midface was stabilized with 13 titanium miniplates, the mandibular body with a 2.0-mm fixation system, and the right orbital floor with a polydioxanone sheet. Nonviable teeth were removed. Because of severe edema and the high risk of iatrogenic facial nerve injury, the right condylar fracture was managed conservatively, and the patient’s habitual occlusion could not be fully restored during the acute operation. Approximately four months after the injury, computed tomography (CT) demonstrated partial osseous bridging and stable residual right zygomatic displacement without signs of an active inflammatory process. **Conclusions**: In devastating intraoral blast injury, airway security, preservation of viable soft tissue, control of contamination, and restoration of a stable skeletal framework take priority over complete acute correction of every fracture and occlusal discrepancy. Staged reconstruction and coordinated psychiatric and internal medicine care are essential components of definitive management.

## 1. Introduction

Firework-related facial injuries are uncommon relative to injuries of the hands and eyes, but direct explosions in or immediately adjacent to the oral cavity may produce disproportionate destruction of both soft tissue and the facial skeleton [1]. Fireworks continue to cause significant facial injuries, and nearly one in five facial injuries from fireworks requires hospitalization [2].

Only a few cases of intraoral firework explosions have been described in the literature, and only two cases of firework-related facial injuries in patients with psychiatric disorders have been reported [3,4]. We found no previous report specifically describing such an injury in a patient with a documented diagnosis of paranoid schizophrenia.

The psychiatric context is integral to the case rather than incidental. Paranoid schizophrenia has traditionally been defined as a subtype of schizophrenia characterized by relatively stable, predominantly paranoid delusions, including persecutory beliefs, usually accompanied by hallucinations, particularly auditory hallucinations, while disturbances of affect and speech, as well as catatonic symptoms, are not prominent [5]. Exacerbations and relapses of psychotic symptoms may be associated with several factors, including non-adherence to antipsychotic treatment, psychosocial stress, and substance misuse. Cannabis and stimulant use has also been associated with the onset or recurrence of psychotic symptoms [6,7]. Clinical manifestations of paranoid schizophrenia can include delusions, auditory hallucinations, disorganized thinking or behavior, negative symptoms, and cognitive impairment [8,9,10]. Second-generation (atypical) antipsychotics remain the cornerstone of pharmacological treatment. If the patient shows little or no response, treatment with first-generation (typical) antipsychotics, clozapine monotherapy, electroconvulsive therapy, or combination therapies may be proposed [11,12]. Schizophrenia is associated with substantial functional impairment, increased rates of relapse and hospitalization, premature mortality, and an elevated risk of self-harm and suicide, with the lifetime risk of death by suicide estimated at approximately 5% [13,14].

Reports published to date emphasize extensive fragmentation of the middle and lower facial thirds, complex oral soft-tissue defects, difficult airway management, and the need for staged reconstruction. The optimal strategy is highly individualized because the extent of tissue devitalization, the reliability of remaining anatomic landmarks, associated injuries, and the patient’s physiological condition may preclude complete reconstruction during the initial operation.

We present a case of massive craniofacial destruction caused by deliberate detonation of a pyrotechnic device in the oral cavity during an exacerbation of paranoid schizophrenia. The report focuses on multidisciplinary airway management, preservation and reconstruction of oral soft tissues, restoration of facial skeletal continuity, and the deliberate surgical compromises required in the acute phase. The manuscript was structured in accordance with the CARE (case report) guidelines [15].

## 2. Case Presentation

### 2.1. Patient Information and Clinical Findings

A 42-year-old man with a diagnosis of paranoid schizophrenia was brought to the emergency department after deliberately detonating a pyrotechnic device in his oral cavity during an acute exacerbation of psychosis. The event occurred in November 2025. The act was regarded as intentional self-harm. The patient’s medical history was significant for arterial hypertension and paranoid schizophrenia. The patient remained under regular psychiatric follow-up at a hospital operated by a private healthcare group.

At presentation, the patient had massive craniofacial trauma and associated polytrauma. He was conscious and fully oriented, with a Glasgow Coma Scale (GCS) score of 15. Communication with the patient was severely limited due to the extent of his injuries, preventing clinicians from obtaining a reliable medical history. His heart rate was 66 beats/min, SpO_2_ was 99%, body temperature was 37.0 °C, and arterial blood gas analysis revealed a pH of 7.35. Active bleeding from the oral cavity and both ears was observed. Additionally, physical examination revealed foreign bodies within the nasopharyngeal and oropharyngeal cavities, as well as bilateral proptosis. Despite the initially preserved oxygen saturation, the patient subsequently experienced an acute decrease in SpO_2_ in the setting of ongoing oral bleeding, raising concern for airway compromise and aspiration of blood. Therefore, oral intubation was performed to secure the airway.

### 2.2. Clinical and Radiological Assessment

**Figure 1:**
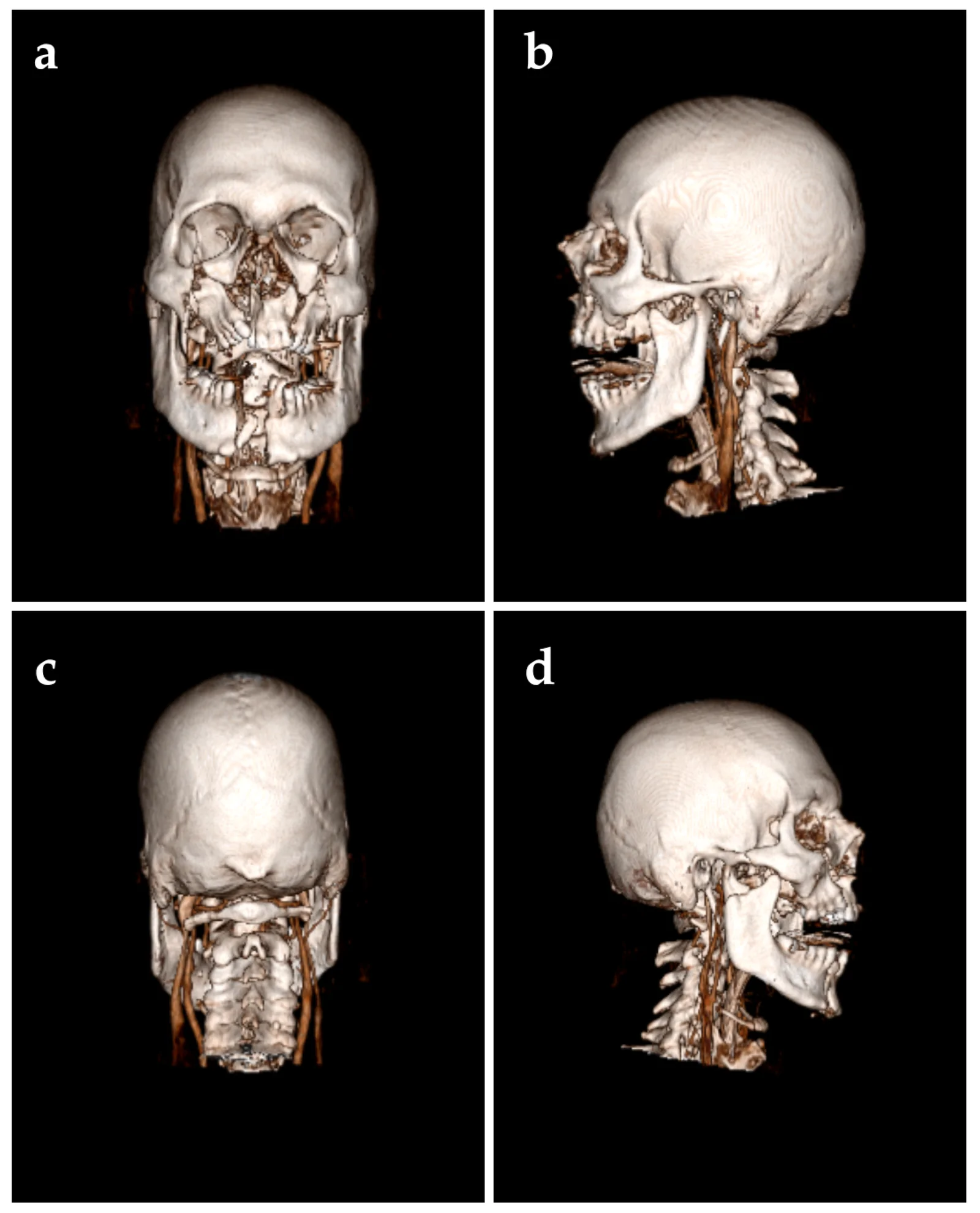
3D CT; (**a**) anterior view, (**b**) left lateral view, (**c**) posterior view, (**d**) right lateral view.

**Figure 2:**
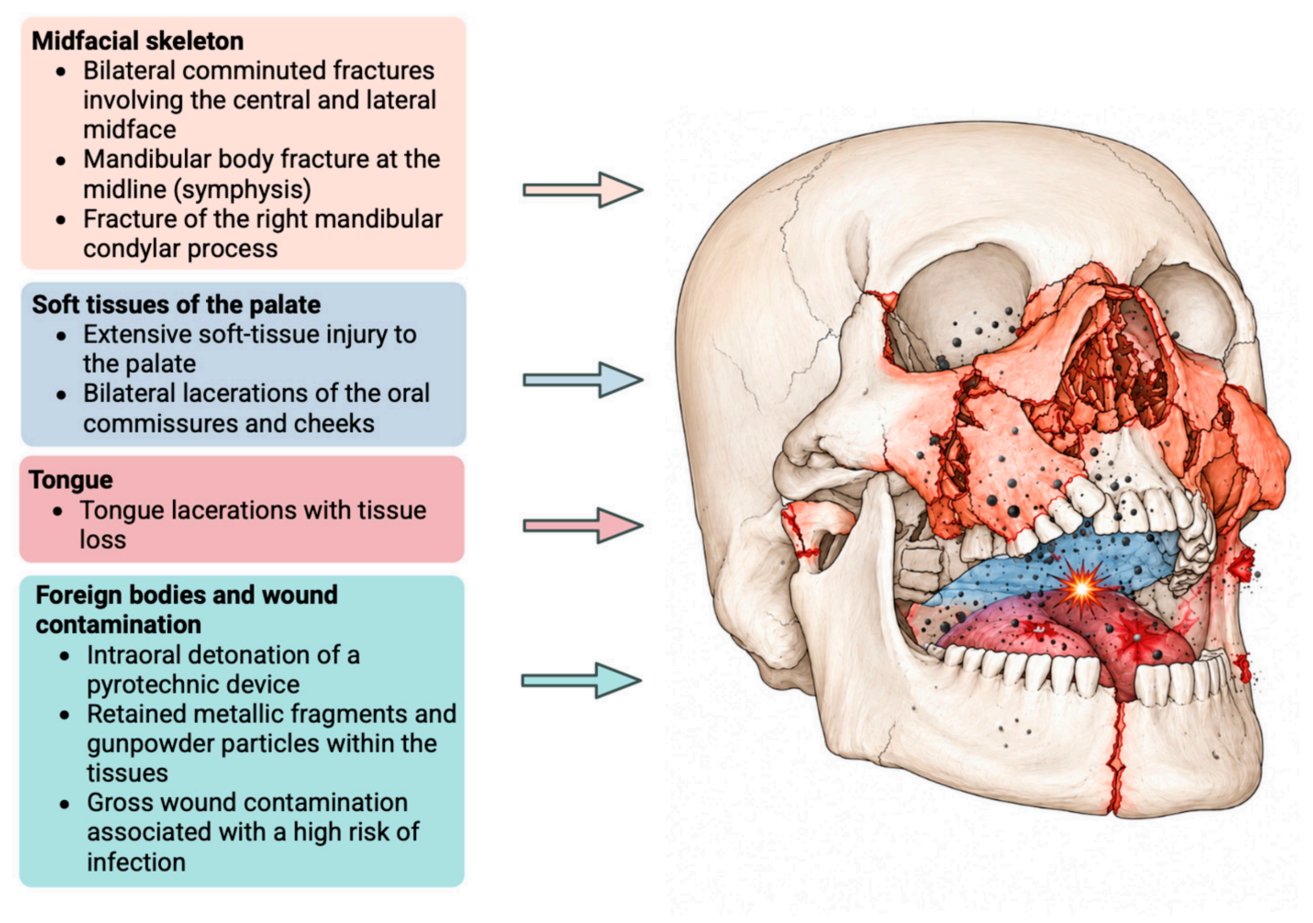
Schematic illustration of extensive craniofacial injuries following intraoral detonation of a pyrotechnic device.

Trauma CT with multiplanar and three-dimensional reconstructions demonstrated extensive bilateral comminution of the middle facial skeleton, involving both the central and lateral midface with marked disruption of normal anatomic landmarks, as well as an associated fracture of the sphenoid bone. A defect of the right orbital floor was also identified. Two mandibular fractures were identified in the lower facial third consisting of a midline body/symphyseal fracture and a fracture of the right condylar process, accompanied by the traumatic damage of teeth 31, 32, 41, and 42 (Figure 1). Soft-tissue injuries were severe and comprised extensive soft-tissue emphysema, a massive palatal wound, and bilateral lacerations with substantial tissue loss involving the tongue and the floor of the mouth. Active bleeding originated from both sides of the palate, the tongue, and the pharynx. Bilateral wounds of the oral commissures and cheeks were also noted. Metallic fragments and pyrotechnic residue were embedded within the tissues, rendering the wounds heavily contaminated (Figure 2). The combination of blast overpressure, thermal injury, and mucosal necrosis substantially compromised standard intraoral access. Concomitant polytrauma findings included compression fractures of the thoracic and lumbar spine (T7, L1, and L4, with L1–L4 involving the transverse processes), a displaced, comminuted, complex fracture of the right acetabulum with fractures of the left superior and inferior pubic rami, multiple bilateral rib fractures, and pulmonary contusion. These extensive associated injuries required immediate multidisciplinary assessment and care from the first day of hospitalization.

### 2.3. Emergency Airway and Soft-Tissue Management

Massive hemorrhage and extensive disruption of the oral cavity prompted conversion from conventional orotracheal intubation to a surgical tracheostomy performed by the otolaryngology team to provide unobstructed access for subsequent reconstruction. In severe maxillofacial trauma, early establishment of a reliable airway is a primary determinant of safe definitive treatment [16,17].

After preparation of the surgical field with an antiseptic solution (Braunol) and ocular protection (Bepanthen), a vertical incision was made in the skin of the neck, followed by layer-by-layer dissection and ligation of the encountered vessels. The thyroid isthmus was divided and ligated to expose the trachea. The anterior tracheal wall was incised between the first and second tracheal rings, and a Björk flap was sutured to the skin. The orotracheal tube was subsequently replaced with a tracheostomy tub, and the cervical wound was closed with adaptation sutures.

The intraoral injuries were then treated surgically under general anesthesia. The previously placed packing and a mobile anterior tooth were removed. Intraoperative examination revealed extensive craniofacial injuries. The tongue showed a laceration of the tongue body approximately 1 cm deep and predominantly oriented along the midline. The tongue was reconstructed with layered closure using 4-0 Vicryl sutures, followed by pharyngoscopy.

One anterior mandibular tooth remained attached only by a small segment of oral mucosa. In addition, there was severe grade III soft-tissue injury accompanied by extensive subcutaneous emphysema. Following multidisciplinary assessment, the medical team decided not to undertake surgical repair of the posterior pharyngeal wall.

A longitudinal laceration involving the hard and soft palate on the left side was identified, with displacement of bony fragments toward the nasal cavity; the nasal bones were also unstable. Open fractures of the maxilla and mandible, consistent with a complex midfacial fracture pattern, were present. The patient was subsequently transferred to the maxillofacial surgery team for definitive reconstruction of the facial skeleton. The maxillofacial and otolaryngology teams operated in a coordinated sequence during the same acute treatment phase.

### 2.4. Skeletal Reconstruction

The maxillofacial team performed a comprehensive 440-min surgical reconstruction. Access to the fragmented midface was achieved through periorbital, paranasal, subciliary, and intraoral approaches. To minimize additional tissue trauma, existing vertical mucosal lacerations caused by the blast and thermal injury were used for intraoral access.

**Figure 3:**
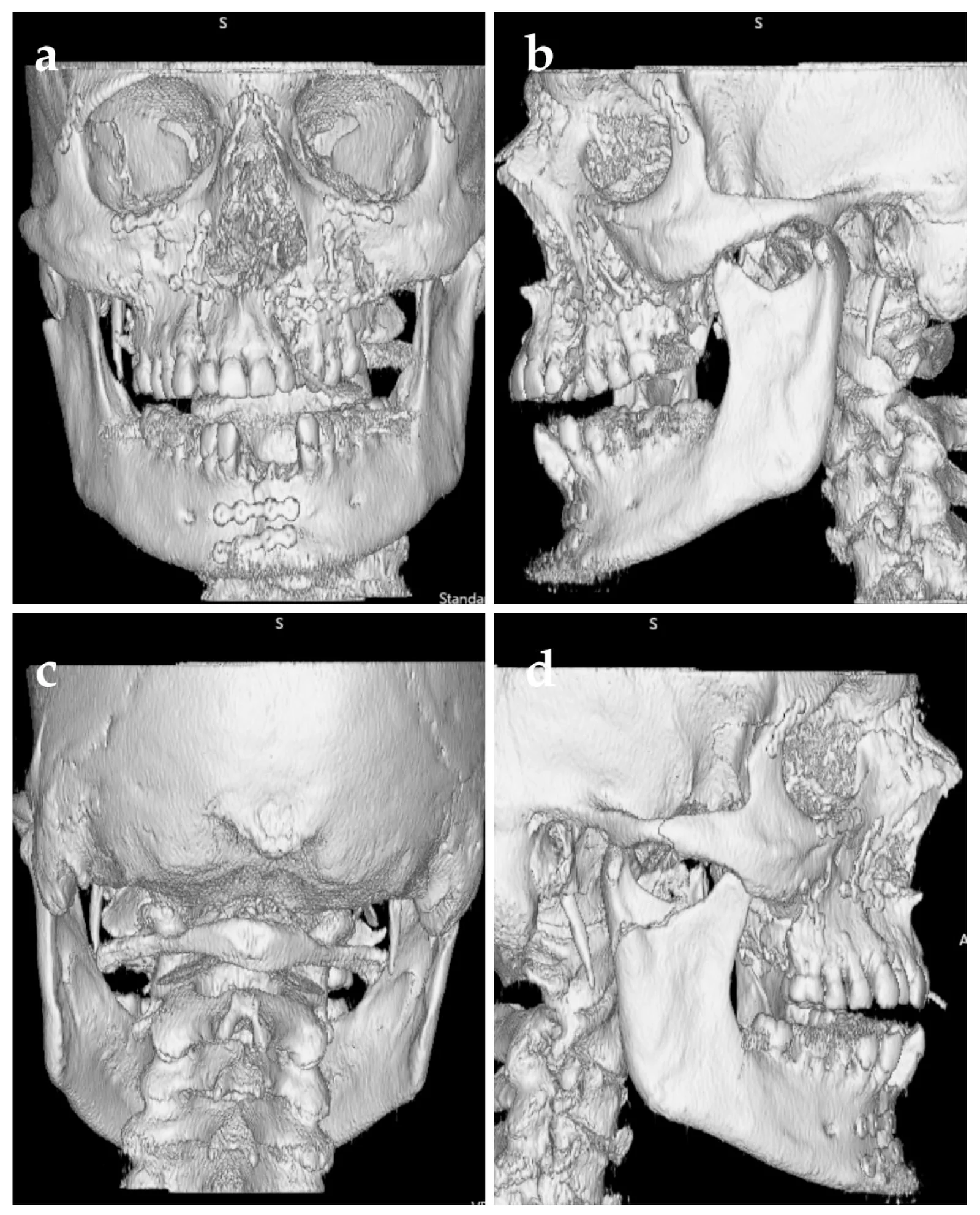
Immediate postoperative 3D CT reconstruction of the facial skeleton, (**a**) Anterior View, (**b**) Left Lateral View, (**c**) Posterior View, (**d**) Right Lateral View.

Reconstruction and reduction of the comminuted midface were achieved using 13 titanium miniplates and 49 screws (Medartis 1.5 HD system; 4- to 5-mm screws) (Figure 3). A polydioxanone (PDS) sheet was placed along the floor of the right orbit to repair the orbital floor defect; the infraorbital nerve was identified intraoperatively and appeared anatomically preserved, without mac-roscopic evidence of transection. Due to severe blast-induced necrosis, the nonviable left maxillary tuberosity was resected along with an impacted third molar. Complete reconstruction of the left palatal shelf was not feasible in the acute setting owing to the extensive soft-tissue deficit. The head of the department determined that acute surgical intervention on the posterior pharyngeal wall and the skull base (sphenoid bone fracture) was deemed unnecessary.

The mandibular body/symphyseal fracture was manually reduced and stabilized using a Medartis 2.0 HD system with two osteosynthesis plates and 5-mm screws. Nonviable teeth posing an unacceptable risk of infection within the fracture line (teeth 31, 32, 41, and 42) were extracted. Open treatment of the right condylar process fracture was deferred. The decision reflected the marked edema around the right temporomandibular joint and the perceived high risk of iatrogenic facial nerve injury. Although open reduction can provide functional benefits in selected displaced condylar fractures, the approach-related neurological risk and the patient’s overall injury burden justified conservative management in this acute setting [18].

Hemostasis was achieved, and airway secretions were managed. Intraoral wounds were closed using 3-0 Vicryl sutures, whereas extraoral incisions were closed in layers using 4-0 Vicryl and 5-0 Monocryl sutures. An orogastric feeding tube was inserted via the oral cavity (facilitated by the palatal defect), and bilateral Merocel nasal packing was applied.

### 2.5. Postoperative Intensive Care

After surgery, the patient remained sedated and mechanically ventilated in the intensive care unit. Management included hemodynamic monitoring, repeated airway assessment, and bronchoscopy with bronchoalveolar lavage to evaluate suspected aspiration-related pulmonary complications. Broad-spectrum antimicrobial therapy with intravenous ampicillin/sulbactam (3 g three times daily) was administered empirically for 11 days to cover mixed oral flora and the heavily contaminated wound. Additionally, tracheal aspirate cultures yielded *Pseudomonas aeruginosa* and *Enterobacter hormaechei* subsp. *hoffmannii*. Bronchoalveolar lavage (BAL) fluid was purulent, with cultures demonstrating *Klebsiella oxytoca*, *Pseudomonas aeruginosa*, and other Gram-negative bacilli. Further microbiological evaluation identified Gram-positive bacilli and Gram-positive cocci. No multi-resistant Gram-negative (MRGN) pathogens or methicillin-resistant *Staphylococcus aureus* (MRSA) were isolated.

Pharmacological thromboprophylaxis with tinzaparin (4500 IU subcutaneously once daily) was initiated during the postoperative period, subject to trauma-related contraindications. Oral intake was withheld, and enteral nutrition was delivered through a gastric tube. The patient remained in the intensive care unit (ICU) for 9 days.

During attempts to reduce sedation, the patient initially exhibited an abnormal emergence pattern characterized by marked agitation, anxiety, and signs of autonomic hyperactivity. These symptoms required pharmacological management with an α-adrenergic receptor agonist to facilitate safe sedation withdrawal and control autonomic stress responses. Following psychiatric consultation, the patient’s psychotropic medication regimen was adjusted according to his current clinical and psychiatric condition. Suicide risk was assessed clinically during psychiatric consultations, including evaluation of suicidal ideation, intent, and the patient’s current mental status. The patient remained under psychiatric supervision throughout hospitalization, with repeated psychiatric assessments. Given the severity of the physical trauma and difficulty communicating with the patient, formal psychotherapy was deferred during the acute phase and was incorporated into the subsequent rehabilitation and mental health care plan at an appropriate facility.

The patient’s long-term antipsychotic regimen included flupentixol 100 mg intramuscularly every 2 weeks and paliperidone palmitate 150 mg intramuscularly every 4 weeks. Levomepromazine, previously prescribed at 25 mg at 08:00, 50 mg at 18:00, and 50 mg at 22:00, was temporarily withheld.

Additional medications comprised bisoprolol 5 mg once daily, torasemide 10 mg once daily, pipamperone 40 mg according to a 0-0-1-1 dosing regimen, quetiapine 200 mg once daily at night, and extended-release biperiden 2 mg once daily. Supportive pharmacotherapy included folic acid 5 mg once daily, potassium chloride 40 mEq with dosing adjusted according to laboratory findings, a vitamin B preparation (Vitamin B Duo) 100 mg once daily, macrogol 3350 mg once daily, metamizole 40 drops four times daily, and naloxegol 25 mg once daily.

During the patient’s stay in the intensive care unit, an ophthalmologic consultation was performed. Ocular contusion (contusio bulbi) was diagnosed, while optic disc edema (papilledema) was excluded. Bilateral ophthalmologic examination demonstrated normal intraocular pressure, progressively resolving eyelid edema and periorbital hematomas, and sutured wounds without signs of infection. Eyelid closure was preserved, and both the corneas and lenses were clear. Pupillary light reflexes were intact, with no evidence of optic disc edema. Examination of the right eye revealed marked conjunctival edema (chemosis). Fundoscopic examination showed a small linear retinal hemorrhage adjacent to a branch of the superior retinal vessel, consistent with ocular contusion. Examination of the left eye demonstrated marked conjunctival chemosis with small conjunctival petechial hemorrhages. The retina remained attached.

Topical ophthalmic treatment was initiated bilaterally with lubricating eye drops (Hylo-Gel) five times daily, ofloxacin eye drops four times daily, and ofloxacin ophthalmic ointment at bedtime. In addition, the right eye was treated with nepafenac eye drops (Nevanac) three times daily. After a 9-day stay in the intensive care unit (ICU), the patient was transferred to the Department of Oral and Maxillofacial Surgery for further treatment and postoperative management.

### 2.6. Postoperative Maxillofacial Care and Transfer to a Rehabilitation Center

During the patient’s stay in the Department of Oral and Maxillofacial Surgery, a percutaneous endoscopic gastrostomy (PEG) tube was placed after 14 days of parenteral nutrition to provide long-term enteral nutritional support. Enteral feeding was initiated with Fres. ENERGY Fibre Easy Bag, administered at 165 mL every 2 h, providing a total daily energy intake of approximately 2000 kcal. Enteral nutrition was well tolerated, with no clinically significant feeding-related complications. The ampicillin/sulbactam therapy initiated in the intensive care unit was continued after transfer to the maxillofacial surgery ward, for a total treatment duration of 11 days.

On the eighth day of hospitalization in the maxillofacial surgery ward, the patient developed catheter-related sepsis. Microbiological examination demonstrated Klebsiella pneumoniae in blood cultures as well as in cultures obtained from the removed central venous catheter. Targeted antimicrobial therapy, selected according to the results of antimicrobial susceptibility testing, was initiated with meropenem 2 g/day for 10 days and lorazepam 1 mg intravenously as needed. A rapid improvement in the patient’s clinical condition was subsequently observed.

In December 2025, the patient was discharged from the hospital and transferred to a specialized rehabilitation center for further multidisciplinary rehabilitation. At the time of transfer, he was conscious, cooperative, and able to maintain a seated position in a chair following progressive mobilization. His cardiovascular and respiratory status remained stable.

As part of the ongoing weaning from mechanical ventilation, the patient was able to breathe spontaneously and effectively through the tracheostomy tube with supplemental oxygen at 1–2 L/min from 07:00 to 19:00, while maintaining compensated gas exchange. During the night, ventilatory support was continued using pressure support ventilation, with a positive end-expiratory pressure of 8 mbar and assisted spontaneous breathing pressure support of 6 mbar.

### 2.7. Follow-Up and Outcomes

**Figure 4:**
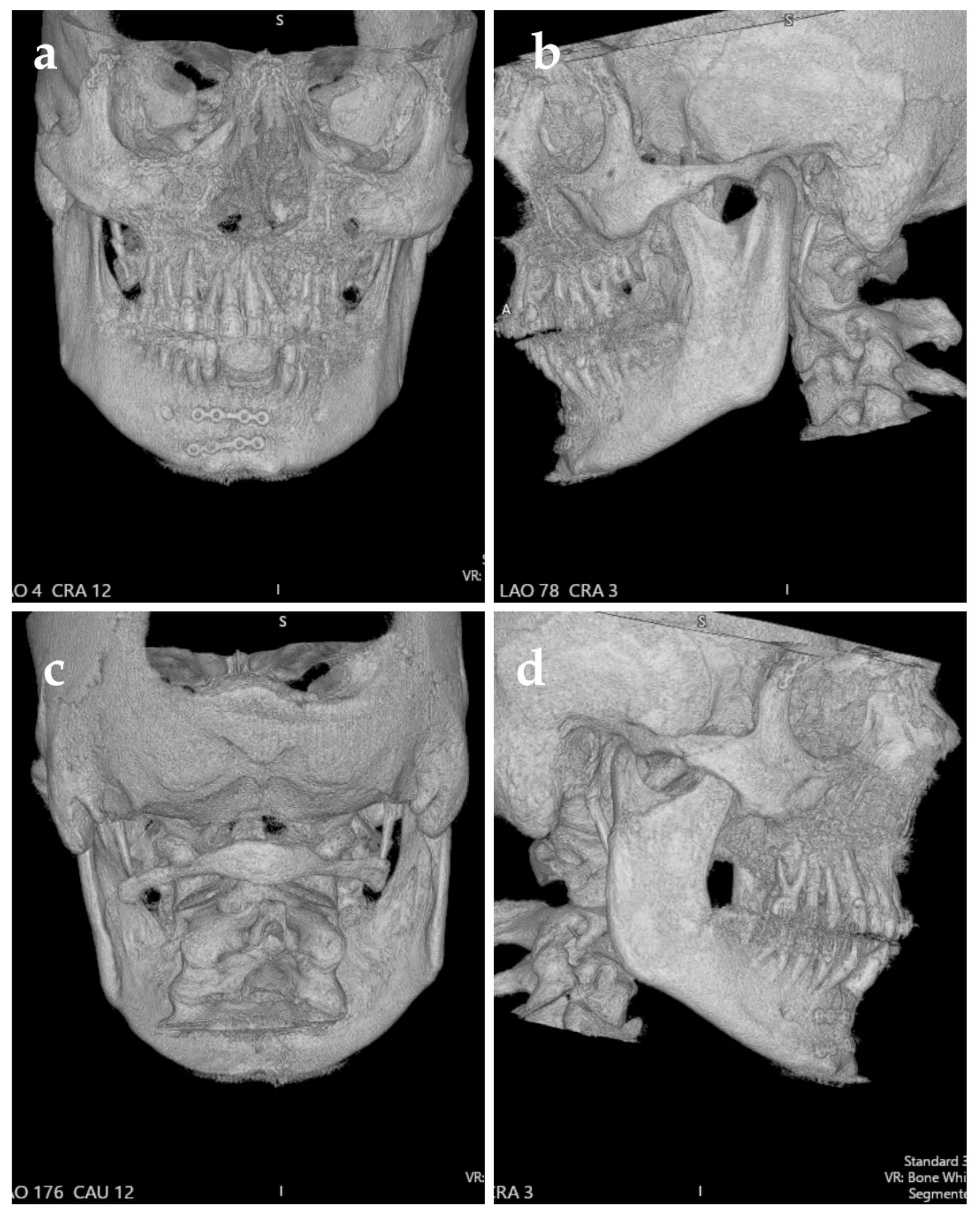
3D CT; follow-up examination four months after the injury, (**a**) Anterior View, (**b**) Left Lateral View, (**c**) Posterior View, (**d**) Right Lateral View.

A follow-up CT examination performed in March 2026, approximately four months after the injury, demonstrated partial ossification across several craniofacial fracture lines. Residual displacement of the right zygomatic bone persisted but appeared stable. Incomplete osseous healing remained visible in selected fracture lines, including the region of the lesser wings of the sphenoid bilaterally (Figure 4). No radiological signs of an active inflammatory process were described. Continued medical surveillance was recommended due to the ongoing progression of ossification.

## 3. Discussion

This case illustrates the extreme destructive potential of an explosion occurring within the oral cavity. The confined location exposes the facial skeleton to an intense pressure wave while simultaneously producing thermal injury, penetrating fragments, and contamination. The resulting pattern-comminution of the midface and mandible combined with palatal, lingual, and perioral soft-tissue loss-is consistent with the limited published literature on intraoral firework explosions [1,3,19,20,21,22]. The additional polytrauma further complicated prioritization and made a single-stage, anatomically complete reconstruction neither realistic nor necessarily safe.

The first decisive intervention was surgical tracheostomy. Severe maxillofacial trauma may rapidly compromise the airway through bleeding, edema, tongue displacement, loss of skeletal support, and aspiration. A secure surgical airway also separates airway management from the operative field and permits prolonged reconstruction without an oral or nasal tube interfering with occlusal assessment and fixation. In the present case, tracheostomy was not merely an anesthetic adjunct; it was a prerequisite for coordinated soft-tissue and skeletal treatment.

The sequence of reconstruction reflected the biological condition of the wound. Repair of the tongue and floor of the mouth before extensive osteosynthesis preserved remaining viable tissue and established a soft-tissue envelope over the reconstructed framework. Blast wounds may contain zones of occult devitalization that evolve over time, and extensive immediate reconstruction can fail when tissue perfusion is uncertain. For this reason, staged reconstruction remains an important principle in major head and neck blast injuries [17].

Panfacial fracture repair commonly uses the mandible and occlusion as reference points, followed by restoration of facial width, projection, and vertical height. However, no universal fixation sequence is superior in every patient; the available stable fragments, degree of comminution, and associated injuries should dictate the order. In this case, the occlusal unit itself was severely disrupted and masked by edema. Attempting to force a definitive occlusal relationship during the initial operation could have propagated errors through the reconstructed midface. Stabilizing the largest viable skeletal segments was therefore a rational damage-control reconstructive strategy.

The decision not to expose and fix the right condylar fracture exemplifies a second deliberate compromise. Open treatment of displaced condylar fractures may improve anatomic reduction and selected functional outcomes, but access can endanger the facial nerve and increase morbidity, particularly in a swollen, traumatized parotid-masseteric region [23]. In a patient with extensive facial wounds, polytrauma, and an already prolonged procedure, conservative management limited additional neurological and soft-tissue risk. The trade-off is the need for structured follow-up of mandibular range of motion, pain, deviation, malocclusion, and temporomandibular joint remodeling.

The four-month CT demonstrated that stabilization allowed partial osseous bridging despite the initial tissue destruction and contamination. At the same time, persistent zygomatic displacement and incomplete healing at selected fracture lines adjacent to the skull base confirmed that acute stabilization was only the first stage. Secondary treatment may include occlusal rehabilitation, management of condylar dysfunction, bone grafting or patient-specific reconstruction of residual defects, scar revision, oral commissure rehabilitation, and dental restoration. These decisions require assessment of imaging findings together with functional outcomes.

## 4. Conclusions

An intraoral pyrotechnic explosion can produce simultaneous airway compromise, complex blast and thermal soft-tissue injury, comminuted panfacial fractures, contamination, and polytrauma. Successful acute management depends on immediate multidisciplinary coordination. In this patient, emergency tracheostomy, preservation and reconstruction of viable oral soft tissue, and stabilization of the largest reconstructible skeletal segments took precedence over complete restoration of habitual occlusion and open treatment of the condylar fracture. The case supports a staged strategy in which the acute objective is survival and establishment of a stable foundation for subsequent reconstruction, followed by functional, dental, and aesthetic rehabilitation. Importantly, the final phase of this staged approach should also incorporate close coordination between post-traumatic rehabilitation and mental health services to ensure continuity of care and address the psychiatric factors that may influence long-term recovery.

## Data Availability

The original contributions presented in this manuscript are included in the references section. Further inquiries can be directed to the corresponding author.

## Abbreviations

The following abbreviations are used in this manuscript:

CT	Computed Tomography
GCS	Glasgow Coma Scale
PDS	Polydioxanone
BAL	Bronchoalveolar lavage
